# Freshwater early life growth influences partial migration in populations of Dolly Varden (*Salvelinus malma malma*)

**DOI:** 10.1007/s00300-021-02870-z

**Published:** 2021-05-28

**Authors:** Christie M. Morrison, Colin P. Gallagher, Keith B. Tierney, Kimberly L. Howland

**Affiliations:** 1 Department of Biological Sciences, University of Alberta, Edmonton, AB T6G 2E9 Canada; 2 Fisheries and Oceans Canada, Freshwater Institute, 501 University Crescent, Winnipeg, MB R3T 2N6 Canada; 3 Fisheries and Oceans Canada, Institute of Ocean Sciences, 9860 West Saanich Road, Sidney, BC V8L 4B2 Canada

**Keywords:** Dolly Varden, *Salvelinus malma malma*, Resident, Anadromy, Life-history, Partial-migration, Otolith back-calculation, Otolith-microchemistry, Growth

## Abstract

Populations of northern Dolly Varden (*Salvelinus malma malma*) exhibit partial seaward migration, yet little is known about this phenomenon in Dolly Varden populations. Our study analyzed data from three different Dolly Varden populations in the western Canadian Arctic in order to determine if: (1) differences in size-at-first seaward migration exist between fish that migrate at early and late ages among populations inhabiting different river systems, and (2) annual growth influences anadromous or resident life history choice. Otolith strontium analysis and back-calculation were used to determine age- and size-at-first seaward migration, respectively. Differences in age- and size-at-first seaward migration were determined across river system and migration age. Back-calculated fish lengths were compared using a mixed effect model to determine how early growth influences migratory tactics (early or late aged smolt, or resident). Our results indicate that fish exhibiting faster early growth migrated in earlier years and at smaller sizes than slower growing fish, however size- and age-at first seaward migration varied by river system. Faster growing Dolly Varden tended to become either residents or early smolts, while slower growth was associated with smolting later in life. This is contrary to life history theory where the fastest growing fish in a population should mature as a resident. Our results indicate factors other than growth may be influencing life history ‘decisions’ in Dolly Varden. Future work on growth efficiencies and metabolic rates is needed to assess how they affect migratory behaviours.

## Introduction

Migration in salmonids is a well-studied phenomenon with the type and extent of migration varying among species and populations. Partial migration (or partial anadromy) occurs when a proportion of the population migrates to more productive habitats (to sea if anadromous) to feed and grow while the balance remains in natal freshwater streams as residents (Jonsson and Jonsson 1993; Chapman et al. 2012). This is observed in species such as Atlantic salmon (*Salmo salar*), brown trout (*Salmo trutta*), Arctic char (*Salvelinus alpinus*) (Klemetsen et al. 2003), brook trout (*Salvelinus fontinalis*) (Theriault and Dodson 2003), and Dolly Varden (*Salvelinus malma*) (Armstrong and Morrow 1980). In populations that exhibit partial anadromy, females tend to dominate the anadromous portion of the population, and males make up the larger proportion of residents (Nordeng 1983; Hutchings and Jones 1998; Jonsson et al. 2001). Partial migration is associated with conditional mating tactics, whereby a single genotype can give rise to either resident or migratory individuals (Jonsson and Jonsson 1993; Hendry et al. 2004), which can spawn together and produce either resident or anadromous offspring (Nordeng 1983; Jonsson and Jonsson 1993; Theriault et al. 2007). In males, reproductive output in the form of fertilization is dependent on the size, condition, and behavior of other males with whom they must compete (Hendry et al. 2004). While larger males are able to maintain a dominant position close to gravid females, and fight off competing males (Fleming and Gross 1994), precocious males that adopt a “sneak spawn” strategy can fertilize a substantial portion of eggs (Theriault et al. 2007) making migration potentially less beneficial to male salmonids.

Many populations that exhibit partial migration are polymorphic, with resident fish having slower growth, reaching smaller sizes, and displaying different morphologies than their migrating counterparts (Jonsson and Jonsson 1993; Rikardsen and Elliott 2000). Anadromous individuals often delay maturity, but benefit from an increased rate of growth, larger size at maturity, and increased reproductive output (Gross 1987; Hendry et al. 2004). However, disadvantages to anadromous behaviour include energy expenditure for migration and osmoregulation, and increased risk of predation during migration and in marine environments.

It is still unclear as to why and when an individual ‘decides’ to adopt a migratory behaviour (Morinville and Rasmussen 2003; Wysujack et al. 2009; Curry et al. 2010), but migratory triggers are generally thought to occur early in life. An individual’s ‘decision’ to migrate may be based on its condition, size, and/or status within the population (Metcalfe et al. 1990; Hendry et al. 2004), and is thought to relate to growth rates and metabolic processes (Jonsson and Jonsson 1993), however the observed patterns are unclear.

Growth is often viewed as a determining factor in a fish’s decision on migration (Hendry et al. 2004). Generally faster growing fish from a population migrate for the first time (smoltification if anadromous) at earlier ages and smaller sizes than their slower growing counterparts (Metcalfe et al. 1989, 1990). Forseth et al. (1999) found that the fastest growing juvenile brown trout in a Norwegian stream migrated at earlier ages and at smaller sizes than older juveniles. Theriault and Dodson (2003) found that larger age-1 individual brook trout migrated at an earlier age and that slow growth was associated with migration later in life (age-2) at a larger size. Likewise findings from studies of resident versus anadromous behaviour are variable; some studies have shown that faster growing fish become residents (Ricker 1938; Thorpe et al. 1998), while others have observed the opposite (Svenning et al. 1992; Rikardsen and Elliott 2000; Olsson et al. 2006). For example, Morinville and Rasmussen (2003) found that resident brook trout were larger by age-2 compared to their pre-migratory anadromous counterparts. In contrast, Rikardsen and Elliott (2000) found that in two populations of Arctic char, the largest fish smolted at age-4, medium-sized fish smolted at age-5, and the smallest fish matured as residents.

Migratory behaviours and size-at-migration not only vary among individuals within a population, but also between years and among populations within the same geographic region (Hendry et al. 2004), indicating that environmental variation may influence migratory behaviour. For example, Jonsson (1985) and Forseth et al. (1999), studying brown trout in different lake systems in Norway, found differences in the average size of fish that chose to migrate to lakes from their natal stream, with large males in the Lake Femund system and medium males in Vangsvatnet Lake system staying as resident, while other brown trout migrated to their respective lakes. Environmental variation occurs in all river systems, which differ in water chemistries, velocity, temperature, migration distance and elevation, area of suitable habitat, species composition, and overall productivity. Systems that vary in environmental conditions tend to produce populations with different migratory tendencies, and also degrees of anadromy (Rikardsen et al. 1997). As an example, Curry et al. (2010) found that brook trout migratory patterns varied from almost complete anadromy to mainly resident in three rivers systems that differed in migration distances, seasonal temperatures, water velocities, and estuary salinities.

Northern form Dolly Varden (*Salvenlinus malma malma*), herein referred to as Dolly Varden, are found in coldwater streams in Alaska, north of the Aleutian Islands, and in the Canadian Arctic, west of the Mackenzie Delta. Dolly Varden exhibit both anadromous and resident life histories within the same genetic population (Harris et al. 2015). Residents are characterized by their small size (< 350 mm), dark colour, visible parr marks, and early maturation. While residents are an important component of these river systems, research has mostly focused on the anadromous life history form; both resident and juvenile (pre-migratory) Dolly Varden in the western Arctic remain largely unstudied (Gallagher et al. 2012, 2019). Therefore, our objectives were to assess populations of Dolly Varden inhabiting three different river systems in the western Canadian Arctic in order to determine if (1) differences in smolt size-at-first seaward migration exist between individuals migrating at an early age vs. those migrating at a late age within and among river systems; and (2) annual growth influences anadromous and resident life history choice within and among river systems.

## Materials and methods

### Study area and sample collection

**Fig. 1:**
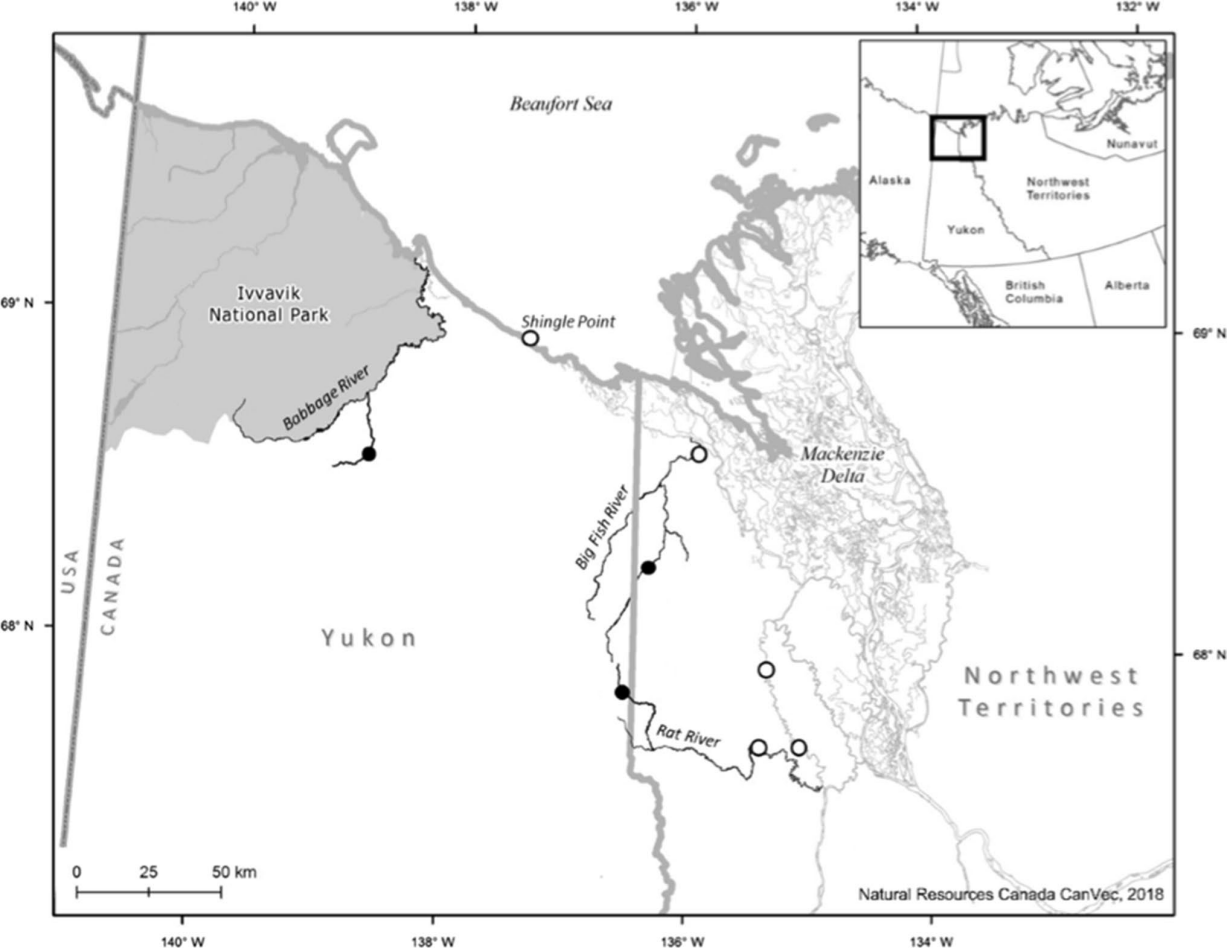
Map of study area illustrating locations where Dolly Varden (*Salvelinus malma malma*) sample collection took place within the Yukon North Slope and Northwest Territories Canada from 2012 to 2015. Black circles represent spawning and/or overwintering sites on the Rat, Babbage, and Big Fish Rivers where resident samples were collected. White circles represent sites where anadromous Dolly Varden were collected during harvest monitoring programs at Shingle Point, the mouth of the Big Fish River, and sites along the Mackenzie River Delta and Rat River

Dolly Varden were sampled from three genetically distinct stocks in the western Canadian Arctic: Rat, Big Fish, and Babbage rivers (Fig. 1). Sampling of anadromous individuals occurred from 2012 to 2015 in conjunction with harvest monitoring programs that occur along the Beaufort Sea Coast at Shingle Point, sites along the Mackenzie Delta and the Rat River, at the mouth of the Big Fish River, and at the spawning/overwintering site in the Big Fish River watershed (Fig. 1). Fish were captured using either 90-, 102-, or 114-mm stretch mesh gill nets with variable lengths and set times, except at the Big Fish River spawning/overwintering site where fish were captured using a 16-m long modified seine net (see Sandstrom et al. 2009). Resident individuals were also sampled with the same seine net (Sandstrom et al. 2009) at the spawning/overwintering sites in the Rat, Big Fish, and Babbage river systems during fall 2012–2016 in conjunction with mark-recapture programs targeting anadromous Dolly Varden. All fish were sampled for fork length (mm), sex, and sagittal otoliths either in the field (anadromous) or later in the lab following storage at − 20 °C (resident). Stock origin for anadromous individuals captured in the marine environment and at sampling sites in the Mackenzie Delta has been determined in previous genetic mixed-stock analysis studies (Gallagher et al. 2020).

### Otolith analysis

Otolith age estimation and thin-section preparation were conducted using methods described by Chilton and Beamish (1982) and Gallagher et al. (2016). Fish were aged using whole sagittal otoliths. Age was estimated by counting the narrow translucent winter growth bands (annuli) on the otolith of each individual. Otoliths were examined by two separate readers with a Quality Assurance and Quality Control (QA/QC) protocol precision target of < 5% difference between readers. Once aged, otoliths were thin-sectioned for otolith strontium (Sr) analysis. Otoliths were embedded in ColdCure™ epoxy resin, and once hardened, were sectioned across the nucleus, perpendicular to the sulcus with a Buehler Isomet slow speed saw (Buehler Ltd., Lake Bluff, Illinois) and diamond wafering blades. Otolith sections were polished with 30-, 9-, and 0.3-μm lapping film and imaged using a Nikon DXM1200 digital camera (Nikon Instruments Inc., Melville, New York) attached to a dissecting microscope.

Otolith Sr analysis was conducted to determine life-time migratory patterns of anadromous individuals (Zimmerman 2005). Sr concentrations in otoliths were analyzed using laser ablation inductively coupled plasma mass spectrometry (LA-ICP-MS) conducted at the University of Manitoba’s Geological Sciences Department (Winnipeg, Manitoba) following preparation methods outlined in Swanson et al. (2010) and Loewen et al. (2015). In brief, otolith sections were embedded in 1-inch Lucite discs using ColdCure™ epoxy, which were polished with 30-, 9, and 0.3-μm lapping film with a final polish on a Buehler polishing wheel (Buehler Ltd., Lake Bluff, Illinois) with 0.05-μm diamond grit paste. Otoliths were ablated from the otolith core to the outer edge of the dorsal lobe, following marked transects overlaid on digital images. Ablation was conducted using a Thermo Finnigan Element 2 High Resolution-Inductively Coupled Plasma-Mass Spectrometer (HR-ICP-MS) (Thermo Fisher, Mississauga, Ontario) in combination with a Merchantek LUV 213 laser ablation system (New Wave Research/Merchantek, Fremont, California) at a 30-μm beam width with a speed of 2 μm s^−1^ and 20-Hz power. A NIST 610 glass standard was run every hour and otolith Sr was standardized to calcium (Ca) in pure aragonite (40.02 wt%) and a NIST610 external standard reference. Concentrations and detection limits were calculated with Iolite software (Paton et al. 2011).

Following LA-ICP-MS analyses, otoliths were imaged and otolith Sr profiles were overlaid on the digital otolith images to align otolith Sr concentrations with otolith annuli (Morris et al. 2005). Age-at- first migration was determined based on increases in otolith Sr at a given annulus as per methods detailed in Howland et al. (2001).

Back-calculation was conducted on otolith cross-sections to determine previous size-at-age for resident fish and up until age-at-first migration for anadromous individuals. Image processing and analysis software, ImageJ 1.44 (National Institutes of Health, Bethesda, MD, USA), was used to measure the distance from the center of the nucleus to each annular increment along the ventral lobe following a linear transect at a 50-degree angle from the sulcus. Based on a linear relationship between fish length and otolith radius in resident fish, the biological intercept model (Campana 1990) was used to back-calculate size-at-age for resident individuals:

$$ L_{i} = L_{c} + \frac{{\left( {O_{i} - O_{c} } \right)\left( {L_{c} - L_{h} } \right)}}{{\left( {O_{c} - O_{h} } \right)}} $$

where *L*_*i*_ is the back-calculated length at a given age, *L*_*c*_ is the fish length at capture, *L*_*h*_ is the fish length at hatch, *O*_*i*_ is the otolith size at a given age, *O*_*c*_ is the otolith size at capture, and *O*_*h*_ is the otolith size at hatch. Otolith size and fish size at hatch was taken from the literature to be 0.06 and 16.5 mm, respectively (Blackett 1968; Armstrong and Morrow 1980; Radtke et al. 1996).

A modification of the biological intercept model, the biological intercept breakpoint model (Morrison et al. 2019), was used to back-calculate size-at-age for post-migratory anadromous individuals due to a decoupling of the fish length – otolith length relationship during first migration:

$$ L_{i} = \begin{array}{*{20}c} {L_{c} + { }\frac{{\left( {L_{c} - L_{j1} } \right)\left( {O_{i} - O_{c} } \right)}}{{\left( {O_{c} - O_{j} } \right)}}{\text{ if }}j \ge O_{j} } \\ {L_{j2} + { }\frac{{\left( {L_{j2} - L_{h} } \right)\left( {O_{i} - O_{j} } \right)}}{{\left( {O_{j} - O_{h} } \right)}}{\text{ if }}j < O_{j} } \\ \end{array} $$

where *L*_*j1*_ is length directly after first migration, *L*_*j2*_ is length directly before first migration, *O*_*j*_ is otolith size at first migration. Individual jump points from pre- to post-migration were determined by individual otolith size at migration. *L*_*j1*_ and *L*_*j2*_ were estimated from population and sex-specific piecewise regressions for the Rat and Babbage river populations, and from a sex-specific piecewise regression based on multiple populations for the Big Fish River population (see Morrison et al. 2019).

### Statistical analysis

A two-way analysis of variance (ANOVA) on size-at-age data followed by a Tukey HSD post hoc test was used to test for differences in size-at-first seaward migration of anadromous males, with river, and age-at-first seaward migration (early or late smolt) as main effects. Normality and equality of variance was determined with a Shapiro–Wilk test and Levene’s test, respectively. Size-at-first seaward migration was based on body size at the time of annulus formation in the year an individual underwent its first migration, and not the specific size at out-migration.

Annual fish growth was determined by subtracting previous size-at-age from size-at-age for the year in question. The mixed effects model by Weisberg et al. (2010) was used to determine growth differences among life history trajectories (early smolt, late smolt, resident) and river systems while controlling for cohort and individual variation in growth. The model is as follows:

$$ y_{cka} = i_{a} + yg_{ck} + yj_{ck} + h_{c + a - 1} + \left( {ih} \right)_{a,c + a - 1} + f_{ck} + e_{cka} $$

where *y*_*cka*_ is the *a*th annual increment for the *k*th fish from cohort *c*, i_a_ is the annual increment at year of life *a*, *yg*_*ck*_ is the life history trajectory for individual *k*, *yj*_*ck*_ is used to represent the river system for individual *k*, *h*_*c*+*a−1*_ represents random environmental effects, *(ih)*_*a,c*+*a−1*_ is a random effects interaction term, *f*_*ck*_ denotes random fish effects, and *e*_*cka*_ represents independent errors with mean zero and common variance *σ*^2^. Linear models with different levels of fixed effects and fixed effect interactions were calculated using maximum likelihood estimation and compared using Akaike information criteria in order to determine the best fit model for describing growth-at-age (Zuur et al. 2009). The best-fit model was then calculated using restricted maximum likelihood estimation and comparisons were conducted with a Tukey HSD post hoc test.

All statistical analyses were completed in R Studio (RStudio Team 2016) and results were considered significant at *α* ≤ 0.05. Only male fish were used in the study due to the limited number of female resident samples (*n* = 2). Sample size includes 99, 67, and 67 anadromous males from the Rat, Babbage, and Big Fish rivers respectively, while resident samples include 73 from the Rat River, 61 from the Babbage River, and 149 from the Big Fish River.

## Results

### Age- and size-at-first migration

Based on age estimates derived from otoliths, and otolith Sr profiles, the majority of male anadromous individuals smolted at age-2 and age-3 in the Babbage River, and age-3 and age-4 in the Big Fish and Rat rivers (Table 1). Early smolts were classified as individuals that migrated at age-2 in the Babbage River and age-3 in the Big Fish and Rat rivers, while late smolts were classified as individuals that migrated at age-3 in the Babbage River and age-4 in the Big Fish and Rat rivers. Age-2 (Big Fish and Rat rivers), age-4 (Babbage River) and age-5 (all systems) were not included in further analyses due to small sample sizes (all *n* ≤ 3; Table 1).

**Table 1:** Frequency (with % in brackets) among age-at- first seaward migration for male Dolly Varden (*Salvelinus malma malma*) from the Rat, Babbage, and Big Fish river stocks in the western Canadian Arctic

River	Age-at-smoltification							
	2		3		4		5	
Rat River	1	(1.0)	34_E_	(34.3)	63_L_	(63.6)	1	(1.0)
Babbage River	36_E_	(53.7)	27_L_	(40.3)	3	(4.5)	1	(1.5)
Big Fish River	1	(1.5)	44_E_	(65.7)	21_L_	(31.3)	1	(1.5)

Age-at-first seaward migration was determined by measuring otolith Sr concentrations among annuli. Samples smolting at earlier (E) and later (L) ages were examined separately among stocks

**Fig. 2:**
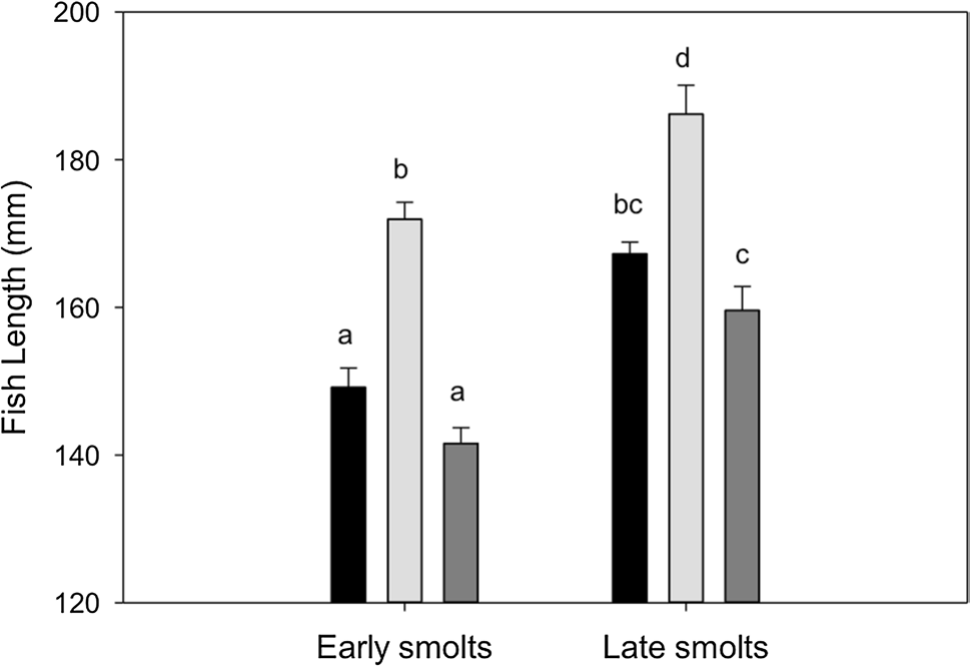
Average size-at-first seaward migration (mm) (± SE) for early and late smolting Dolly Varden (*Salvelinus malma malma*) from the Rat River (black), Big Fish River (light grey), and Babbage River (dark grey) stocks in the western Canadian Arctic. Letters represent significant differences (*p* < 0.05)

Based on the two-way ANOVA, average size-at-first seaward migration differed among the three populations (*F*_2,206_ = 61.2, *p* < 0.001) and between early and late smolts (*F*_1,206_ = 67.2, *p* < 0.001), with early smolts being smaller than late smolts. The river*age-at-first seaward migration (early or late smolt) interaction was not significant (*F*_2,206_ = 0.5, *p* = 0.624). Differences in average size-at-first seaward migration (± SE) were observed between Babbage and Big Fish river (*p* < 0.0001), Babbage and Rat river (*p* = 0.007), and Big Fish and Rat river (*p* < 0.0001), with Big Fish River Dolly Varden being the largest (172 ± 2 mm), followed by Rat River (158 ± 2 mm), and Babbage River (151 ± 2 mm) (Fig. 2). The smallest size-at-first seaward migration was 113 mm from an age-2 smolt from the Babbage River, while the largest was 209 mm from an age-4 smolt from the Big Fish River (Table 2).

**Table 2:** Average size-at-first seaward migration (mm) (± SE) with range (mm) in brackets for early and late male Dolly Varden (*Salvelinus malma malma*) smolts from the Rat, Babbage, and Big Fish river stocks in the western Canadian Arctic

River	Average size-at-smoltification (mm)					
	Early smolt			Late smolt		
Rat River	149	± 3	(123–183)	167	± 2	(139–199)
Babbage River	142	± 2	(113–166)	160	± 3	(115–194)
Big Fish River	172	± 2	(143–202)	186	± 3	(149–209)

### Life history and river differences

The best model for describing annual growth (i.e., lowest AIC value) included age, life history trajectory (early smolt, late smolt, resident), river system, and interaction terms between age and life history trajectory, and between age and river; all while controlling for variation among cohorts and individuals (Table 3). This model indicated that annual juvenile growth differed significantly among ages (*F* = 3267.4, *p* < 0.0001), life history trajectories (*F* = 25.9, *p* < 0.0001), and river systems (*F* = 78.2, *p* < 0.0001). It also indicated that growth differences among the rivers and among life history trajectories depended on age (*F* = 34.7, *p* < 0.0001 and *F* = 7.6, *p* < 0.0001, respectively).

**Table 3:** Model parameters of annual growth in Dolly Varden (*Salvelinus malma malma*) from the Rat, Big Fish, and Babbage river stocks in the western Canadian Arctic from mixed effect models

Model parameters	AIC	ΔAIC
Growth = age + life history + river + age*life history + age*river + life history*river + age*life history*river + (cohort + individual)	11180.51	9.12
Growth = age + life history + river + age*life history + age*river + life history*river + (cohort + individual)	11173.11	1.72
Growth = age + life history + river + age*life history + age*river + (cohort + individual)	11171.39	0
Growth = age + life history + river + age*life history + life history*river + (cohort + individual)	11328.41	157.02
Growth = age + life history + river + age*river + life history*river + (cohort + individual)	11200.56	29.17
Growth = age + life history + river + age*life history + (cohort + individual)	11325.55	154.16
Growth = age + life history + river + age*river + (cohort + individual)	11199.18	27.79
Growth = age + life history + river + life history*river + (cohort + individual)	11379.83	208.44
Growth = age + life history + river + (cohort + individual)	11378.97	207.58

Random effects are in parentheses with * denoting an interaction effect. AIC values were determined from maximum likelihood estimation

**Fig. 3:**
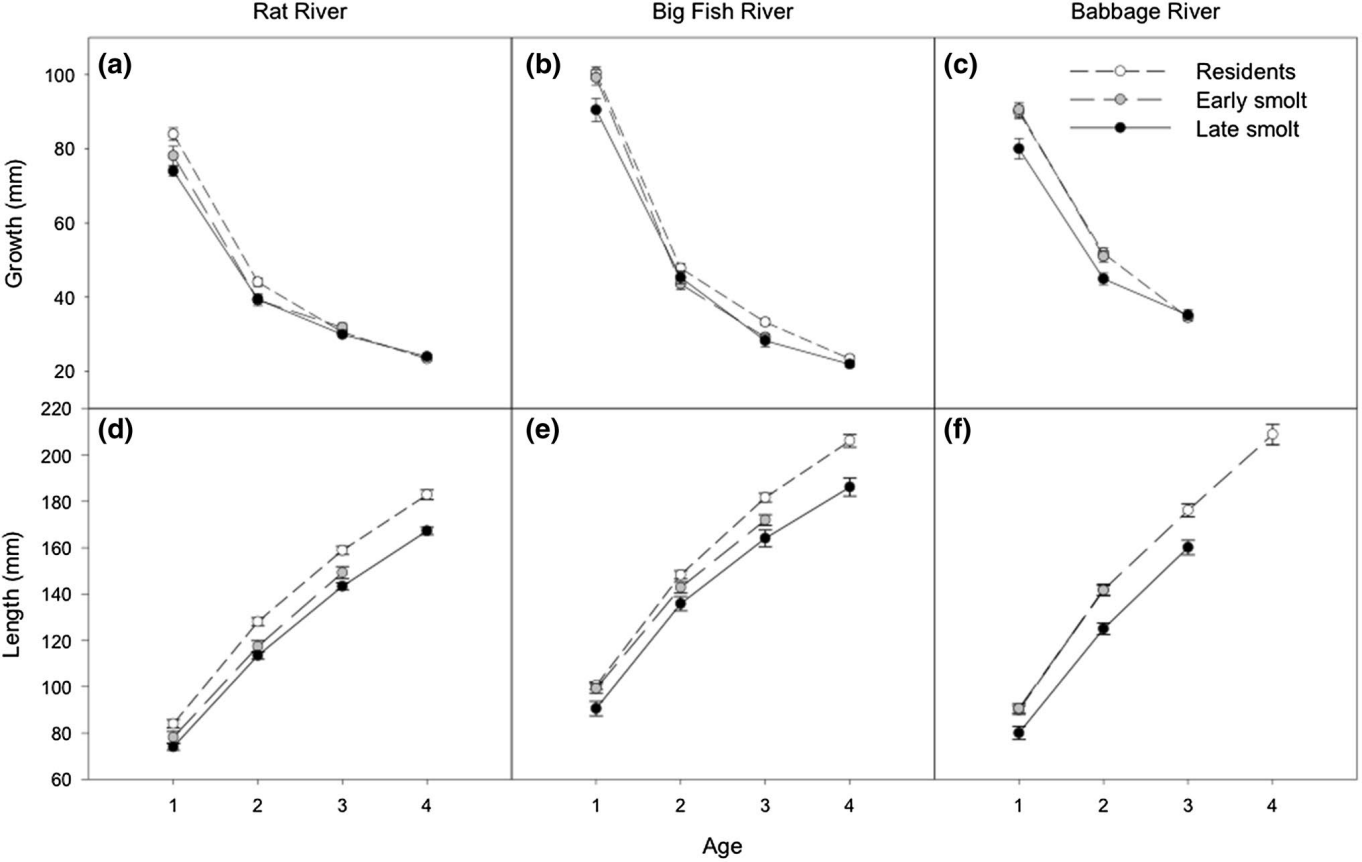
Average (± SE) **a**–**c** growth-at-age (mm), and **d**–**f** size-at-age (mm) for resident, early smolting, and late smolting Dolly Varden (*Salvelinus malma malma*) males from the Rat, Big Fish, and Babbage river stocks in the western Canadian Arctic

When comparing growth at age-1 to age-4 in life history trajectories (Table 4; Fig. 3) we found that residents typically showed similar growth to early smolts, while late smolting fish had the slowest growth in all river systems by age-1. Statistical differences in growth were found at age 1 between late smolts and early smolts (*t*_1466_ = 6.1, *p* < 0.0001), and between late smolts and residents (*t*_1272_ =  − 8.4, *p* < 0.0001) in all rivers, with late smolts having slower growth in both cases. At age-1 there was no statistical differences in growth between resident and early smolting individuals (*t*_1475_ = − 1.8, *p* = 0.8140), however, a trend of higher average growth in residents relative to early smolts was observed on the Rat River at age-1 (Fig. 3). At age-2, late smolts had lower growth compared to residents (*t*_1272_ = − 3.3, *p* = 0.0426) in all three river systems (Table 4; Fig. 3). There were no statistical differences in growth found between early and late smolts (*t*_1466_ = 0.437, *p* = 1.0) and between early smolts and resident Dolly Varden (*t*_1475_ = − 3.1, *p* = 0.0799) at age-2. However, we noted trends suggesting increased growth occurred at age-2 in residents vs early smolts in the Rat River and in early smolts vs late smolts in the Babbage River (Fig. 3). No statistical differences in growth were observed at age-3 and age-4, although a trend towards increased growth in age-3 residents compared to both early and late smolts was observed for the Big Fish River (Fig. 3).

**Table 4:** Statistical results from the Tukey HSD post hoc tests on growth differences in Dolly Varden (*Salvelinus malma malma*) life history trajectories (resident, early smolt, late smolt) and river systems from age-1 to age-4

		Age 1			Age 2			Age 3			Age 4		
		*t*	*df*	*p*-value	*t*	*df*	*p*-value	*t*	*df*	*p*-value	*t*	*df*	*p*-value
*Life history trajectories*													
Resident	Early smolt	− 1.8	1475	0.8140	− 3.1	1475	0.0799	− 1.2	1477	0.9893	–	–	–
Resident	Late smolt	− 8.4	1272	< 0.0001	− 3.3	1272	0.0426	− 1.8	1263	0.8032	− 0.4	1333	1.0000
Early smolt	Late smolt	6.1	1466	< 0.0001	0.4	1466	1.0000	0.4	1472	1.0000	–	–	–
*River systems*													
Rat River	Babbage River	5.2	1459	< 0.0001	7.4	1459	< 0.0001	2.8	1454	0.1702	2.4	1434	0.3880
Rat River	Big Fish River	14.6	1476	< 0.0001	3.8	1476	0.0098	0.3	1479	1.0000	− 0.8	1452	0.9998
Babbage River	Big Fish River	− 8.6	1479	< 0.0001	3.9	1479	0.0057	2.5	1478	0.3245	3.0	1448	0.1084

When comparing life history trajectories across ages (Fig. 3), we found that residents had the largest overall size-at-age based on the observed growth. However, there was considerable overlap with residents and early smolts, especially in the Babbage River at all ages, and at age-1 in the Rat and Big Fish rivers (Fig. 3). Late smolts consistently had smaller size-at-age compared to early smolts and residents.

**Fig. 4:**
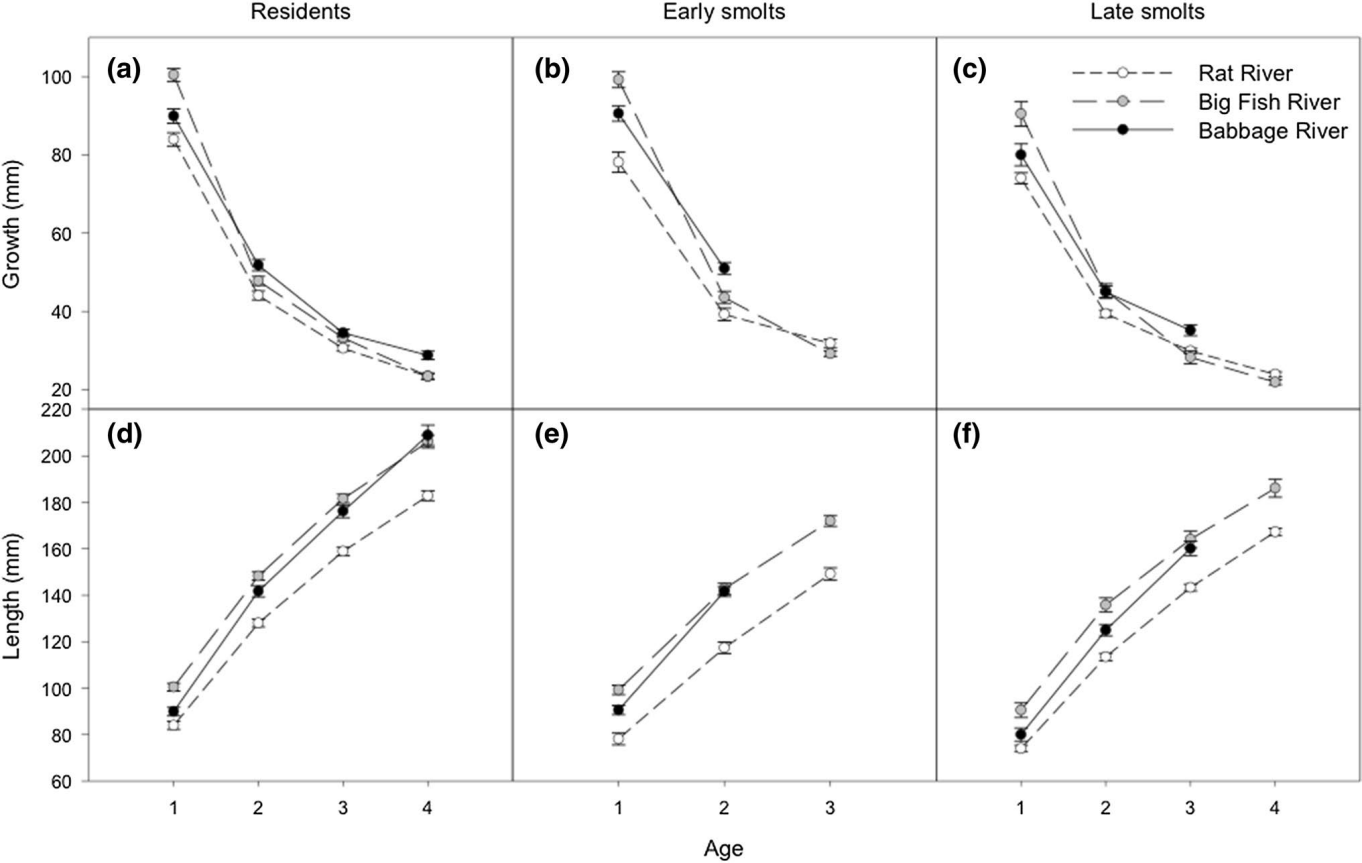
Average (± SE) **a**–**c** growth-at-age (mm), and **d**–**f** size-at-age (mm) for Dolly Varden (*Salvelinus malma malma*) males from the Rat, Big Fish, and Babbage river stocks in the western Canadian Arctic comparing residents, early smolts, and late smolts

When comparing growth at age-1 to age-4 among river systems (Table 4; Fig. 4) we found that Rat River Dolly Varden had the lowest growth at ages-1 and -2 while Big Fish River and Babbage River Dolly Varden exhibited the most growth at age-1 and age-2, respectively. Statistical differences were found at age-1 (Table 4), with Dolly Varden from the Big Fish River exhibiting larger growth than those from the Babbage River (*t*_1479_ = − 8.6, *p* < 0.0001), and Rat River (*t*_1476_ = 14.6, *p* < 0.0001), while Babbage River Dolly Varden exhibited larger growth compared to those in the Rat River (*t*_1459_ = 5.2, *p* < 0.0001). Statistical differences were found at age-2 (Table 4), with Babbage River Dolly Varden exhibiting larger growth than those from the Big Fish River (*t*_1479_ = 3.9, *p* = 0.0057), and Rat River (*t*_1459_ = 7.4, *p* < 0.0001), while again Big Fish River Dolly Varden exhibited larger growth than those from the Rat River (*t*_1476_ = 3.8, *p* = 0.0098). However, we observed similar growth trends in late smolts at age-2 in the Big Fish and Babbage rivers (Fig. 4). No statistical differences in growth were observed at age-3 and age-4 (Table 4). However, a trend of higher growth for age-3 late smolts and age-4 residents in the Babbage River was observed (Fig. 4).

Based on the growth trajectories, Rat River Dolly Varden have the smallest overall size-at-age compared to Dolly Varden from the Big Fish and Babbage rivers (Fig. 4). While Babbage River size-at-age was initially smaller than Big Fish River, increased growth between age-1 and age-2 shows Babbage River reaching comparable size trajectories with Big Fish River Dolly Varden by age-2 for early smolts and age-3 for resident and late smolts (Fig. 4).

## Discussion

Our study demonstrated that growth in early life influenced migration tactics of Dolly Varden. Results showed that there was a difference in size-at-first seaward migration between Dolly Varden that migrated as early smolts and those that migrated as late smolts, with early smolts migrating at smaller sizes compared to late smolts. We also found differences in age- and size-at-first seaward migration among river systems with fish from the Babbage River migrating at earlier ages and smaller sizes compared to those from the Big Fish and Rat rivers. Dolly Varden from the Big Fish River tended to migrate at the largest sizes. Reconstructing early life growth patterns and size-at-age of Dolly Varden using otoliths demonstrated that the fastest growing fish in the examined populations tended to become either resident or early smolts while slower growth was associated with anadromy and smolting later in life. We also found that Dolly Varden growth differed among river systems, with fish from the Rat River exhibiting the slowest growth among systems.

The pattern of size differences between early and late smolting Dolly Varden observed in our study is consistent with Dolly Varden populations in Kamchatka (Gruzdeva et al. 2017), and numerous studies on other salmonids such as Arctic char (Svenning et al. 1992; Rikardsen and Elliott 2000), brook trout (Morinville and Rasmussen 2003; Theriault and Dodson 2003), brown trout (Jonsson 1985; Forseth et al. 1999), and Atlantic salmon (Metcalfe et al. 1988; Jonsson et al. 1998). Anadromy is often considered a size-dependent tactic since a certain threshold size is needed for osmoregulation (Conte and Wagner 1965; McCormick and Saunders 1987). Even after reaching osmoregulatory threshold sizes, smaller-sized individuals have higher costs associated with energy expenditure for migration and osmoregulation, as well as an increased susceptibility to size-selective mortality through predation (Hendry et al. 2004). Therefore, benefits exist for slower growing fish to delay anadromous migration until the following year.

In our study, we observed differences in age- and size-at first seaward migration; fish that had the least growth by age-1 migrated at age-3 in the Babbage River, and age-4 in the Big Fish and Rat rivers. Differences in age- and size-at-first seaward migration may be due to genetic differences among populations. Evidence suggests that there is a genetic basis for the timing of within-year smolt migration (Nielsen et al. 2001; Stewart et al. 2006; Achord et al. 2007; Thorstad et al. 2012), which may extend to genetic differences between years. Okland et al. (1993) suggested that fish with lower metabolic rates have increased difficulties with osmoregulation in marine environments. While the extent of metabolic differences among the populations examined in this study is unknown, it is likely that there are genetic differences due to local adaptations.

Adaptation to local environmental conditions in the Big Fish River has possibly led to a slight increase in size-at-first seaward migration due to increased costs associated with smoltification. While the Big Fish and Babbage rivers have a similar migration distance to the sea (131 km and 145 km respectively), they differ in relation to freshwater environments. The Babbage River exhibits winter water temperatures around 4 °C and low mineral content (Sandstrom et al. 1997), while the Big Fish River is characterized by warm winter water temperatures ranging from 8 to 16 °C, a high mineral content, and low dissolved oxygen (DO) levels compared with other river systems in the region (Mochnacz et al. 2010). The process of smoltification requires sufficient DO (Stefansson et al. 2008), and while low DO levels in the Big Fish River do not appear to hamper the onset of smoltification, low DO levels may play a role in the delayed smoltification observed for the Big Fish River. Evidence also suggests that water quality issues such as low pH level, increased heavy metals, contaminants, etc. can influence smoltification (McCormick and Saunders 1987; Stefansson et al. 2008). Other plausible reasons for this size discrepancy in smoltification is that the Big Fish River may experience relatively higher predation rates while at sea (Gallagher, unpublished data), thus larger fish may have a selective advantage during first migration compared to smaller individuals, leading to a genetic shift in size-at-first migration in this particular population.

Similarities were found between size-at-first migration for Dolly Varden from the Rat and Babbage rivers, with Rat River individuals taking an extra year to reach similar migrating sizes compared to individuals from the Babbage River. Delayed smoltification in the Rat River compared to the Babbage appears to be a function of size and growth rather than fish age, suggesting that the Rat River may have a lower productivity compared to the Babbage River.

Growth comparisons between resident and anadromous Dolly Varden, indicates that juvenile growth did not differ between residents and early smolts. These are consistent with observations of Dolly Varden in Kamchatka (Gruzdeva et al. 2017), female Dolly Varden from the western Canadian Arctic (Gallagher et al. 2019), and findings in other salmonids by Morinville and Rasmussen (2003) and Theriault and Dodson (2003). However, compared to early smolts, residents from the Rat and Big Fish rivers had a trend towards increased size at age-1 to age-3 on the Rat River, and age-2 and age-3 on the Big Fish River. Similar to our findings on the Rat and Big Fish rivers, Morinville and Rasmussen (2003) found that though no statistical differences were observed between resident and anadromous growth rates, there was a trend of larger size-at-age in resident fish compared with their anadromous counterparts.

When comparing growth among river systems we found that Big Fish River Dolly Varden, while initially having the largest growth in age-1, experienced less growth in subsequent years compared to those from the Babbage River, which led to similar size-at-age between Dolly Varden from these two systems. Sandstrom (1995) determined that egg size from the Big Fish River tend to be larger than those from the Babbage and Rat rivers, which could lead to larger fish emerging from the gravel. Warmer groundwater temperatures in the Big Fish River (Mochnacz et al. 2010) could also lead to earlier emergence and thus a longer growing season for young fish. Subsequent higher growth rates in the Babbage River and comparable size-at-age after age-1 could indicate higher freshwater growth potentials and productivity in the Babbage River.

Simulations by Hutchings and Jones (1998) indicate that increases in early growth rates in salmonids favours early maturation (i.e., residency) instead of anadromy. Resident Dolly Varden have been known to mature as young as age-2, while maturation in anadromous individuals often does not occur until a few years after first migration (age-5 to -6) (Gallagher et al. 2018). The higher growth rate in residents, especially compared to late smolts, and observed milting in residents sampled in this study indicate that these ‘larger’ residents had reached maturation. According to Thorpe (1994) maturation at a young age is the goal of an individual in order to maximize fitness. Therefore, once a threshold size has been reached, an individual should mature and become a resident. This coincides with general life history theory about maximizing fitness by maturing at a young age. Since size in males does not necessarily bring about an advantage in fitness potential (Hendry et al. 2004), migration becomes a less desirable tactic. The model of maturation versus migration described by Thorpe et al. (1998) is slightly more complex with numerous thresholds being met at given time periods. Those individuals who do not meet certain maturation size thresholds can choose to migrate and benefit by having a better competitive advantage for reproduction once mature, due to an increase in growth obtained during marine migration. However, this tactic carries the risk of increased predation and delays maturation by a considerable number of years. It is possible that there is within-year growth differences between early smolt and resident Dolly Varden thus having residents reach thresholds not attainable by early smolts. These differences would not be observable through back-calculated growth rates, which show similar overall annual growth.

Our findings of similar growth and size-at-age between resident and pre-migratory juveniles indicates that growth is not the only driver of life history ‘decisions’. Studies have suggested that other factors, such as metabolic rate, social status, growth efficiencies, and lipid stores may also contribute to migratory ‘decisions’ (Metcalfe et al. 1989, 1995; Rowe et al. 1991; Metcalfe 1998; Jonsson and Jonsson 2003; Morinville and Rasmussen 2003). Morinville and Rasmussen (2003) studied brook trout and found that although residents had increased size-at-age, future anadromous individuals consumed 1.4 times more food than residents but had lower growth efficiencies and higher total metabolic costs. The pre-migratory anadromous brook trout in their study allocated 38–53% of their energy towards metabolism compared to 25–45% by residents. Forseth et al. (1999) studied freshwater migration and residency of brown trout and determined that while there were no differences in growth, consumption rates were higher in migrating trout compared to residents, and metabolic costs were considerably higher in migrating individuals. Morinville and Rasmussen (2003) proposed that the variation in metabolic rates seen in resident and anadromous individuals could either be results of differences in activity levels, or differences in standard metabolic rate. Aggressive behaviour is often linked to early migrating individuals as opposed to residents or late smolts (Metcalfe and Thorpe 1992; Metcalfe et al. 1995; Lahti et al. 2002). Metcalfe (1998) suggested that dominant individuals tend to be more aggressive, which leads to energetic costs and a decrease in growth potential. Metcalfe et al. (1995) also demonstrated that fish with lower standard metabolic rates migrate later in life compared to early migrants, indicating that there are likely metabolic differences between residents and early smolts as well. Therefore, fish that migrate early, and do not remain as stream residents, may become growth limited and thus migrate to more productive habitats in order to satisfy metabolic costs.

Our study suggests that early growth is an important factor in determining life history trajectories in Dolly Varden, particularly between early and late smolts. Numerous factors can influence growth rate, such as egg size, time of hatch, time of emergence, first feeding, habitat availability, and climate variability. Any change in these factors has the potential to influence a population’s growth rate and life history trajectories. While Dolly Varden in our study demonstrated differences in annual juvenile growth between early and late smolts and between anadromy and residency, there was still considerable overlap in growth-at-age, indicating that other factors influence an individual’s migratory ‘decision’, such as growth efficiencies and metabolic rate. Further research on the contribution of growth efficiencies and metabolic rate to life history ‘decisions’ is needed in order to assess whether these other factors influence life history trajectories for Dolly Varden.
